# Adopting Soft Logic to enhance an Adizes tool: A comprehensive approach to represent the evaluation of management styles

**DOI:** 10.1371/journal.pone.0327672

**Published:** 2025-08-01

**Authors:** Sigal Kordova, Ron S. Hirschprung

**Affiliations:** Department of Industrial Engineering and Management, Faculty of Engineering, Ariel University, Ariel, Israel; Agricultural Research Organization, ISRAEL

## Abstract

Management skills assessment has become increasingly complex in today’s digital age, as these skills are multi-dimensional and require a nuanced approach. Beyond evaluating each dimension independently, it is crucial to consider the tension and balance between them, as they often exist in inverse proportions. To address this complexity, this study introduces a methodology based on Soft Logic, a mathematical theory designed to represent realities that are not clear-cut, such as conflicting traits. This approach is particularly suited to the assessment of managerial skills. The research focuses on the Adizes Management Style Questionnaire, a widely recognized tool that evaluates four distinct managerial styles: Producer, Administrator, Entrepreneur, and Integrator. Unlike traditional assessments that focus on isolated behaviors, the proposed method highlights the dynamic interplay between these styles. By incorporating Soft Logic, the framework provides a more flexible and comprehensive evaluation, capturing both dominant traits and the tensions between them. This methodology was tested in an empirical study with 40 participants and offers organizations deeper insights into leadership dynamics, aiding in recruitment, leadership development, and team composition. Although developed with the Adizes tool, the methodology can be adapted to other managerial assessment tools and potentially extended to other multi-dimensional domains.

## 1. Introduction

In today’s fast-paced and complex business environment, the success of organizations hinges not only on strategic decision-making, but emphasis is also placed on effective leadership and management styles. As the nature of work and organizational structures evolve, traditional models of management are being challenged, necessitating more comprehensive approaches to evaluating and guiding leaders. Geopolitical contexts further complicate this dynamic, significantly influencing management and organizational practices, and presenting unique challenges and opportunities for leaders navigating complex environments [1].

Leadership and management styles play a critical role in the functioning and success of organizations, particularly in navigating complex social dynamics. The rapidly evolving environment and the need to navigate an open market, technological advancements, and the increasing demands of customers for quality and service have fundamentally reshaped the managerial role. Traditional models, focused primarily on control, stability and incremental improvement, often assume predictable environments and clear hierarchies. However, these assumptions have become outdated in light of the fluid, decentralized, and innovation-driven reality of the 21st century [2,3]. In this complex world, managerial responsibilities must shift from rigid task delegation to dynamic sense-making, learning orientation, and adaptive leadership. Managers can no longer rely solely on static procedures and – instead must develop the capacity to respond to ambiguity, manage contradictions, and lead across functional and cultural boundaries [4,5]. For example, overemphasis on task outcomes can result in a dissatisfied team, while focusing solely on people development might yield minimal tangible achievements. Consequently, modern managers must continuously learn and adapt, balancing seemingly opposing or complementary roles. It has shown that leadership and management training is a key ingredient in the health system, and this was demonstrated in low-income countries [6]. Mitchell and Boyle [7] examine how inspirational leadership drives team innovation by cultivating a positive mood, with professional salience serving as a key moderating factor. By promoting a shared vision and encouraging positive emotions, inspirational leaders support flexible thinking and collaboration. However, the impact of these efforts hinges on professional salience, which enables positive mood to effectively harness professional diversity. The study underscores the significance of team composition and targeted leadership training in multidisciplinary environments. The contemporary approach to management necessitates proficiency in fulfilling “both-and” roles rather than relying on the traditional dichotomy of “either-or” [5]. Effective leadership directly influences the organizational climate, employee motivation, and overall productivity [8]. An essential aspect of understanding and optimizing leadership styles is their alignment with the organizational culture, including factors such as Cultural Fit. As Nieminen, Biermeier-Hanson, and Denison [9] argue, leadership effectiveness significantly depends on the alignment between a leader’s behavioral tendencies and the organization’s cultural context. Their Leader–Culture Fit Framework highlights how such alignment fosters synergy and organizational success while misalignment can lead to tensions and reduced performance. Previous researches have explored various aspects of management and leadership styles, which are closely related but not identical. Management styles may be defined as: “the principles that underline the methods, abilities and techniques managers use in handling situations and expressing leadership within an organization. Generally speaking, managerial styles are polarised between autocratic and permissive, although each style has its own subtleties” [10]. Lopatta, Tideman, Böttcher, and Wichern’s [11] study provides a comprehensive review on the impact of management styles on firms’ strategic decisions and overall performance. The authors suggest that future research should delve deeper into the aggregated characteristics at the board level and explore whether managers’ individual styles shape corporate decisions or managers are selected based on characteristics that align with a firm’s existing strategy. In this context, ethnic and cultural diversity within a workforce often introduces unique managerial challenges and opportunities. The presence of diverse cultural backgrounds requires tailored approaches to management, fostering innovation while addressing potential conflicts. These dynamics further highlight the importance of considering cultural and contextual factors when evaluating and developing management styles [1].

Given that a management style inherently involves the ability to influence others, it is intrinsically linked to leadership, which Yukl [12] defines as the “process of influencing others to understand and agree about what needs to be done and how to do it, and the process of facilitating individual and collective efforts to accomplish shared objectives”, therefore, as mentioned above leadership is closely related to management.

While the project management theory often overlooks the leadership style as a success factor, general management theory emphasizes its importance for organizational performance [2]. Management style theories have also evolved from McGregor’s Theory X (the belief that workers lack ambition and internal motivation) and Theory Y (that assumes that employees want to work and to take responsibility) to the more recent Z style, which considers cultural influences on management approaches [13,14].

Leadership styles are vital in driving organizational management changes, with various styles identified as promoters of change processes, including authoritarian, transformational, laissez-faire, servant, transactional, democratic, strategic, bureaucratic, consultative, and participative leadership [15]. Effective leadership is considered essential for successful organizational change and maintaining competitiveness in dynamic markets [15]. In organizations, leadership is characterized by individuals who set objectives, initiate productive initiatives, and hold the authority to direct and influence employees. Different leaders adopt varied approaches: some focus on efficient execution of tasks, others on strategic decision-making, while still others prioritize innovation and ideation that inspire their followers [8]. Leadership is thus seen as a key factor in driving employee productivity and shaping shared organizational norms [16].

According to Bass and Bass [17], “In the business and industrial sector, leaders’ effectiveness is measured objectively by their organizational units’ profit, profit margin, sales increase, market share, return of investment (ROI), unit productivity, cost per item product, and cost relative to budgeted cost”, and also factors such as safety and voluntary turnover, as well as the attitudes and perceptions employees hold toward their manager [18]. It is important to recognize that a leader’s approach to these outcomes is deeply influenced by their unique interpretation of situations, which is shaped by personal background, experiences, and values – leading to diverse decision-making styles within organizations [8,11,19].

Given the demands and complexity of the management figure, a sophisticated tool is required to assess managers’ abilities to balance conflicting needs and adapt to various challenges [20]. Such tools support organizations in assembling balanced leadership teams where different managerial styles complement one another, leading to a comprehensive and well-rounded management approach. The insights derived from these assessments enable organizations to align managerial skills with business needs, develop targeted training programs, and enhance overall leadership effectiveness. Therefore, tools like the Adizes Management Styles Questionnaire have become increasingly relevant [21]. This diagnostic tool assesses managerial skills and competencies, focusing on four key styles: Producer, Administrator, Entrepreneur, and Integrator (commonly known as the PAEI framework). Each style embodies distinct managerial capabilities essential for organizational success. The questionnaire aims to identify a manager’s strengths and potential gaps in these areas, providing insights into their natural approach to leadership and management tasks. According to the raw results of the questionnaire, an analysis highlights the level to which a manager excels to achieve the desired results (Producer), ensuring structured processes (Administrator), innovating and adapting (Entrepreneur), and fostering teamwork and cohesion (Integrator).

The Adizes Management Style Questionnaire, widely recognized for assessing managerial competencies, relies on four singular measurements that may overlook the nuanced tensions between these types in an individual. This has become crucial in today’s organizations characterized by complexities of leadership roles, and highlights the limitations of traditional evaluation methods. While management and leadership are often viewed as overlapping concepts, and indeed are closely related terms, scholarly literature consistently distinguishes between their core functions. Management typically focuses on maintaining structure, implementing plans, and overseeing operational systems to achieve defined objectives. In contrast, leadership centers on guiding change, shaping vision, and mobilizing people through inspiration and alignment. Although both roles involve working with people and influencing behavior, management emphasizes control and efficiency, whereas leadership emphasizes direction and transformation. Recognizing this distinction is essential for understanding how different managerial styles – such as those indicated within the Adizes framework – encompass both administrative coordination and visionary guidance [22,23]. For example, consider a manager whose leadership style combines the characteristics of an Integrator (I) and an Entrepreneur (E). While the Integrator focuses on team cohesion and collaboration and the Entrepreneur drives innovation and strategic vision, this type of a manger lacks the ability to execute decisions. On the other hand, a manager with a strong Administrator (A) profile excels in executing decisions and maintaining operational order but might struggle with generating new ideas or fostering a creative environment. In many organizations, the perceived ideal manager would embody the “both-and” approach—possessing both entrepreneurial creativity and administrative efficiency. However, in practice organizations often face non-ideal trade-offs: they must choose between candidates (for promotion or recruitment) who might be strong in one style but lack in another. For instance, one candidate may demonstrate strong visionary thinking (E) but lack the discipline to follow through (A), while another may offer stability and order (A) but lack the agility to innovate (E). These imperfect choices reflect the complexity of real-world managerial roles, where individuals rarely exhibit fully balanced competencies across all dimensions. This reality calls for a major two-dimensional assessment, where one dimension is the rank of the types – indicating the level of each style, and the other dimension is the tensions between the types’ ranks – indicating the level of balance. To address this issue, in this research we adopted the theory of Soft Logic (described in section 3), a mathematical approach designed to accommodate ambiguity and multi-dimensional analysis. By representing the interpretation of the Adizes questionnaire results with Soft Logic, it is possible to capture not only the dominant types, but also the intricate, often conflicting dynamics between them. In the above examples, the use of Soft Logic allows a more nuanced representation of such candidates’ evaluations by quantifying their capacity to navigate these dual roles. Instead of forcing a deterministic classification, Soft Logic accommodates the complexity of these traits. Accordingly, this research seeks to answer the following question: How can managerial styles be more accurately represented and evaluated by integrating Soft Logic into the Adizes framework, especially in capturing the multi-dimensional nature of this tool and the tensions between the conflicting dimensions.

This enriched representation allows organizations to better understand the managerial tendencies of individuals, leading to more accurate assessments and tailored development strategies. This paper proposes that adopting Soft Logic alongside the Adizes methodology (or another selected evaluation tool) can enhance its diagnostic accuracy, providing a more comprehensive approach for evaluating management styles in the complex realities of modern organizations. The contribution of this study not only enhances the diagnostic potential of the Adizes tool but also offers a theoretical advancement in the representation of complex managerial personal traits.

## 2. Literature review

Leadership and management are dynamic and multifaceted fields that have undergone significant transformations in recent decades. The evolution from traditional command-and-control models to more flexible, adaptive, and people-oriented approaches reflect the increasing complexity and uncertainty in organizational environments. The importance of understanding leadership and management styles cannot be overstated, as they directly influence organizational culture, employee satisfaction, productivity, and overall organizational success [24].

One of the pioneering scholars in leadership and management studies is Bass [25] who significantly advanced the study of this discipline by distinguishing between transformational and transactional leadership styles. **Transformational leadership** is characterized by a leader’s ability to inspire and motivate followers to achieve more than what is typically expected of them. Transformational leaders are often visionaries who foster innovation, encourage creativity, and seek to empower their followers by aligning the followers’ goals with the broader organizational vision. Bass identified four key components of transformational leadership: a) Idealized influence, where leaders act as role models, b) Inspirational motivation, where leaders articulate a compelling vision, c) Intellectual stimulation, where leaders encourage creativity and critical thinking and d) Individualized consideration, where leaders attend to the individual needs of their followers. In contrast, **Transactional leadership** is based on a system of rewards and punishments. Transactional leaders focus on the exchange process between leader and follower, where compliance and performance are rewarded, while failure to meet expectations results in corrective action or punishment [26]. This style of leadership is more focused on maintaining the status-quo and ensuring that tasks are completed efficiently. Bass described two main components of transactional leadership: a) Contingent reward, where rewards are provided in exchange for meeting objectives, and b) Management by exception, which involves monitoring to reveal deviations from standards and intervening when necessary. Bass’ research indicated that while both leadership styles can be effective, transformational leadership is often associated with higher levels of employee satisfaction, commitment, and performance. Transformational leaders are seen as more effective in driving organizational change and fostering a positive work environment, whereas transactional leaders are more effective in stable environments where tasks are routine and clearly defined.

Based on Bass’s insights of the effectiveness of different leadership styles, Adizes [21] introduced a management model that identifies four key roles: *Producer* (*P*), *Administrator* (*A*), *Entrepreneur* (*E*), and *Integrator* (*I*):

a) **The Producer (P)**: Producers are practical and task-oriented, focusing primarily on executing tasks with strong emphasis on content over form. They are dedicated and hardworking, often working long hours to ensure tasks are completed effectively. Producers tend to handle tasks independently, while preferring direct involvement rather than delegation. They are short-term thinkers and perform well under pressure, making them highly effective in crises. This style aligns with the operational management role emphasizing productivity and efficiency.
b) **The Administrator (A)**: Administrators are characterized by their linear, purposeful, and organized approach to management. They place high priority on efficiency and methodology, ensuring that processes are streamlined and effective. Administrators focus on the procedures and methods by which tasks are accomplished rather than just the outcomes. Administrators prefer to be completely wrong rather than partially right, indicating their commitment to precision and order. In higher organizational roles, administrators often serve as CFOs or heads of operations, where their skills in structuring and systematizing are invaluable.
c) **The Entrepreneur (E)**: Entrepreneurs are visionary and creative, who are able to foresee trends and opportunities before others. They are energetic and filled with innovative ideas, often bringing a fresh perspective to the organization. However, their focus on ideation can sometimes lead to a lack of execution ability, and they may be seen as somewhat dreamy or detached from the practicalities of implementation. Entrepreneurs are often argumentative, constantly challenging the status quo and encourage new ways of thinking.
d) **The Integrator (I)**: Integrators play a crucial role in unifying the organization by bridging the different management styles. They are adept at understanding and navigating organizational politics, listening to various perspectives, and aligning with the majority opinion. Integrators can see the overall organizational picture, and foster cohesion and collaboration among team members. They excel in mediating conflicts and calming disputes, and in ensuring a harmonious work environment.

Adizes emphasized that the right blend of these styles is essential for fostering both stability and innovation in organizations. In other words, when evaluating a manager, not only the level of each style should be considered, but also the tensions between these styles, which derives the balance (in an inverse ratio, the greater the tension the lower the balance and vice versa). Therefore, the isolated evaluation of each style is not sufficed, and **balancing** these management styles within an organization contributes significantly to its overall health and performance. Each style brings unique strengths that, when harmonized, enhance the organization’s ability to adapt, innovate, and thrive. Müller [27] addresses the requirement of a balanced leadership style in project management, and also refers to the shifting of leadership approaches over time. Zhang and Fong Foo [28] reviewed the field of balancing leadership by analyzing classic Chinese as well as Western perspectives. Their findings show that it has become imperative for leaders to practice balanced leadership. McDermott et al. [29] emphasize the critical role of managers in shaping psychological contracts that align with an organization’s HR strategy. These contracts, representing the shared expectations between employees and the organization, are significantly influenced by leadership style. Transformational leadership fosters long-term alignment with organizational values, while transactional leadership is better suited for achieving short-term performance objectives. This strategic alignment promotes higher levels of employee commitment, satisfaction, and overall organizational success.

Based on the concept of leadership styles, Wang et al. [30] examine the impact of servant leadership, highlighting its ability to foster team reflexivity, a process where team members collectively evaluate their goals and operational strategies. This collaborative reflection promotes employee creativity by nurturing an environment of trust, empowerment, and mutual support. Their study underscores the significance of leadership approaches that not only enhance performance, but also cultivate a culture of collaboration and innovation. Lundqvist et al. [31] conducted a systematic review of learning-oriented leadership, examining its relationship with individual, group, and organizational learning. Their findings highlight that leadership behaviors, such as intellectual stimulation and promoting team reflexivity, can directly and indirectly foster workplace learning.

Building on different frameworks, Goleman [32] introduced six leadership styles based on emotional intelligence: Coercive, Authoritative, Affiliative, Democratic, Pacesetting, and Coaching. Goleman argues that emotionally intelligent leaders are more effective because they can tailor their approach to the emotional and situational needs of their teams. For instance, the **Authoritative** style, which is visionary and motivational, is best suited for providing direction during periods of change, while the **Affiliative** style, which emphasizes relationships and harmony, is ideal for repairing a fragmented team. Lewin, Lippitt, and White [33] identified three primary leadership styles: Autocratic, Democratic, and Laissez-faire. Their research demonstrated that the **Democratic** style, which involves participative decision-making, often results in greater group satisfaction and higher productivity. The **Autocratic** style, which is more directive, can lead to efficient decision-making but may stifle creativity and lower morale. The **Laissez-faire** style, where leaders provide minimal direction, was found to be less effective, often leading to confusion and inefficiency.

In recent years, research has expanded to include the influence of globalization, technological advancements, and cultural diversity on management styles. For example, Avolio, Walumbwa, and Weber [34] explored the concept of *Authentic Leadership*, where leaders are encouraged to be self-aware, transparent, and ethical. This style has been linked to higher employee engagement and trust, particularly in organizations undergoing significant change. Yukl [35] introduced the concept of *Flexible Leadership*, highlighting the importance of a leader’s ability to adapt their style according to the demands of the situation. This approach aligns with the dynamic nature of contemporary organizations, where change is constant, and leaders must be agile in their decision-making. Chua et al. [36] examined the role of cultural intelligence in leadership. They found that leaders with high cultural intelligence are better able to navigate the complexities of managing diverse teams, leading to improved communication and collaboration across cultural boundaries. Their research underscores the growing importance of cultural adaptability in global organizations. As organizations increasingly integrate digital technologies, it has become imperative for leaders to cultivate competencies in digital literacy and innovation management. The transformational leadership approach described above, emphasizes innovation and continuous improvement, and has been identified as particularly effective in facilitating digital transformation [37,38].

A summary of the key leadership models in the literature presented in Table 1. It includes: the references that cite the models, key features, the conceptual connections to the Adizes PAEI framework, and the degree of relevance—classified as *Supports* (the model reinforces core principles of Adizes), *Corresponds* (the model conceptually aligns with specific PAEI roles), and *Relevant* (the model contextually contributes but lacks direct alignment).

**Table 1:** Comparative overview of leadership models in relation to the Adizes PAEI framework.

Model/ Theory	Key Features	Connection to Adizes	Relevance to Adizes Model
Transformational vs. Transactional Leadership [25]	Transformational: vision, innovation, empowerment. Transactional: rewards/punishments, task control.	Transformational aspects align with Entrepreneur/Integrator; transactional aligns with Administrator/Producer.	Corresponds
Leader–Culture Fit Framework [9]	Effectiveness depends on alignment between leadership behaviors and organizational culture.	Conceptually related to how Adizes types align with organizational context.	Relevant
McGregor’s Theory X, Y, Z [13,14]	X: directive control. Y: empowerment and trust. Z: culture-sensitive integration.	Provides background assumptions about motivation relevant to Adizes style effects.	Relevant
Lewin’s Leadership Styles (Autocratic, Democratic, Laissez-faire) [33]	Autocratic: directive. Democratic: participatory. Laissez-faire: hands-off.	Captures core tensions similar to Adizes trade-offs between control and flexibility.	Corresponds
Goleman’s Emotional Intelligence Styles [32]	Six styles based on emotional intelligence (e.g., authoritative, affiliative, coaching).	Illustrates emotional behavior range relevant to interpersonal aspects of Adizes types.	Relevant
Authentic Leadership [34]	Leaders are authentic, self-aware, ethical, and promote trust and engagement.	Authenticity and balance resonate with Integrator and Entrepreneur aspects.	Supports
Flexible Leadership [12]	Leaders adapt style to situation demands; dynamic and responsive.	Directly parallels Adizes idea of adaptability across roles.	Supports
Servant Leadership [30]	Leader supports others’ growth, promotes trust and team reflection.	Supports the Integrator’s focus on team development and cohesion.	Supports
Learning-Oriented Leadership [31]	Focus on fostering learning at the individual, team, and organization levels.	Aligns with Adizes’ learning and role flexibility over time.	Supports
Adizes PAEI Model [39]	Producer, Administrator, Entrepreneur, Integrator – balanced execution, order, innovation, and cohesion.	Focal point of this paper; Soft Logic enhances its dynamic evaluation.	---

While there are different approaches to evaluate managerial skills, there is consensus that such an evaluation should be multi-dimensional, where each dimension should not be isolated from the others. Manuel, Esteve, & van Witteloostuijn [40] referred to the Multifactor Leadership Questionnaire (MLQ), and even claimed that second-order factors should be used in multi-dimensional models to describe leadership styles. The study of management styles continues to evolve, reflecting the complexities of modern organizations. These developments highlight the importance of adaptability and continuous learning, suggesting that the most effective managers are those who can navigate diverse challenges and foster a positive, innovative, and inclusive work environment.

## 3. Soft Logic background

Pure mathematics can be deterministic, however life is usually not. The difference lays not only in the probabilistic nature of most events, but also in their vogue behavior. For example, in many cases we cannot clearly state that one candidate for a managerial job is better than the other, because each has his or her own merits. Moreover, considering the domain of this research, evaluation of management skills has two major dimensions: the rank of each ingredient that forms the management style; and the tensions between the ingredients. While one candidate for example can have a higher rank, the other one may have a lower tension between the skills, and therefore will be more balanced. Situations like this introduce a dilemma, and Soft Logic is aimed to address these issues.

The Soft Logic theory was developed and introduced by Klein and Maimon [41], mainly to provide a mathematical representation to situations that are vogue, unclear, paradoxical and so on. For example, a very common phenomenon is the privacy paradox, where the concern for an individual’s right to privacy does not line up with the behavior. Soft Logic was adopted in this case to represent the paradox as detailed below [42].

To address the abovementioned aims, Soft Logic differs from classical mathematics in a very central principal: The zero number is no longer a singular value, but may have an infinite number of values. Therefore, the statement: ∀ a,b∈ℝ : a0=b0 is not necessarily true under Soft Logic which introduces a new term: soft zero. Soft zero is noted as $\overset{―}{0}$, and its range of values is received by multiplying it with a classical mathematics scalar. Now, if a≠b  then $a\overset{―}{0}\neq b\overset{―}{0}$, e.g., in classic math 2·0=3·0, but in Soft Logic 2·0≠3·0.

A soft number comprises two parts: a real part which is a classical mathematics number (noted by $\overset{―}{1}$), and a soft part which is a soft zero number (noted by $\overset{―}{0}$). The need to split a number into two parts is relevant when there is an intrinsic difference between the content they represent. For example, in the above-mentioned phenomenon, the privacy paradox, two major variables may be observed with relation to an individual privacy. One is the Privacy Concern, which is the amount of privacy the user decided to preserve. For example, an e-commerce user states that even for a significant discount personal information will not be disclosed. The other, Privacy Behavior, is the amount of privacy the user actually preserves. For instance, even when a minor discount is given, an e-commerce user agrees to the disclosure of personal information. These two variables are not principally different – therefore, Soft Logic is not required. However, when dealing with the paradoxical situation that stems from the gap between privacy concerns and privacy behaviors, the user’s technological literacy should also be considered. This is due to the fact that a user with low literacy most probably is unaware of his or her actions, therefore, the paradox can be explained. A new size is now introduced, which is the multiplication of the gap and the user’s literacy (reversed). This size measures the magnetite of the paradox, and has different semantics and units than the privacy concern and behavior. In soft Logic, the privacy concern and behavior, which are relatively solid and tangible, may be represented by the real part of the soft number, and the paradox, which is relatively elusive, by the soft part. Thus, the ‘entire picture’ may be encapsulated by a single number, without any data loss, and with the ability to manipulate mathematically. This capability has practical implications, e.g., users’ privacy issues may be considered more easily when designing an application that encompasses a privacy violation potential. Another example, which is closely related to the current research subject, is a decision-making process where there are some options, and several measurable parameters that support the decision (e.g., when considering which product to purchase, the price, performances, reliability, maintained… of several optional products). Each parameter represents a solid and tangible size, therefore it can be represented as the real part of the soft number. On the other hand, trade-offs between the parameters, e.g., when parameter A is better for product 1, but parameter B is better for product B, may be represented as the soft part. In this case, the facts are the real part, and the dilemma is the soft part. A common way to handle this situation is to weigh each parameter, which will yield a singular number to ranks each option. Nonetheless, this approach suffers from a significant data loss and may reduce the accuracy of the decision, e.g., when there are critical values for certain parameters.

Like any other theory, Soft Logic is also based on axioms [43], which are detailed below:

### Axiom 1 – Distinction:

if a≠b  then $a\overset{―}{0}\neq b\overset{―}{0}$

The substance of this axiom is the declaration that there are different values of zeros, therefore, this axiom is the heart of Soft Logic, and its existence actually “stretches” the zero number from a singular point to an infinite axis. Moreover, the axiom derives an order between soft zeros, so that: if a<b then $a\overset{―}{0}<b\overset{―}{0}$.

### Axiom 2 – Addition:

$a\overset{―}{0}+b\overset{―}{0}=(a+b)\overset{―}{0}$.

This axiom defines how the addition of two soft zeros is defined, and together with the already known definition of the addition of classic numbers, provides the basis for the addition operator of soft numbers. The inspiration for this axiom stems from classic algebra, i.e., the distributive property: a·(x+y)=a·x+a·y [44].

### Axiom 3 – Nullity:

$a\overset{―}{0}*b\overset{―}{0}=0.$

This axiom creates a transformation between a soft zero and a real zero. The multiplication of two soft zeros produces a real zero. This axiom was inspired by the dual number property: $\varepsilon^{2}=0$ [45].

### Axiom 4 – Bridging:

$a\overset{―}{0}⊥b\overset{―}{1}$ exist

A bridge exists between a soft zero and a real part. This axiom practically connects the ‘worlds’ of the real number and the newly introduced soft zero, thus, a soft number can be formed. The bridge is noted by ⊥. This axiom is greatly related to the complex numbers theory, where the real part of a number and the imaginary part (a multiplication of a scalar with $i=\sqrt{-1}$) can be bridged to form a complex number: a+bi [46].

### Axiom 5 – Non-commutativity:

$a\overset{―}{0}⊥b\overset{―}{1}\neq b\overset{―}{1}⊥a\overset{―}{0}$.

This axiom differs Soft Logic from the most classical math. Nonetheless, some preliminary cases can be found where there is a lack of commutative rings in some algebraic structures, for example a·b≠b·a in a non-abelian group [47].

Now, based on the above definitions and axioms, a soft number can be defined as:


$$a\overset{―}{1\;}\dot{+\;}b\overset{―}{0}\;\;(a,b\in \mathbb{R})$$

and the set of soft numbers is $SN=\left\{a\overset{―}{0}\dot{+}b:a,b\;\in R\right\}$.

Given the definition of a soft number, basic mathematical operators can also be defined, as described in Table 2.

**Table 2:** Definition of mathematical operators of Soft Logic.

Operator	Definition
Addition	$\left(a\overset{―}{0}∔b\right)+\left(c\overset{―}{0}∔d\right)=(a+coverline0∔(b+d)$
Multiplication	$\left(a\overset{―}{0}∔b\right)\times (c\overset{―}{0}∔d=(ad+bc)\overset{―}{0}∔(bd)$
Inverse of a number	${\left(a\overset{―}{0}\dot{+}b\right)}^{-1}=\left(-\frac{a}{b^{2}}\overset{―}{0}\dot{+}\frac{1}{b}\right)$ when b≠0,
n-th power	${\left(a\overset{―}{0}\dot{+}b\right)}^{n}=n{ab}^{n-1}\overset{―}{0}\dot{+}b^{n}$ where $n\in {\mathbb{N}}^{+}\;,\;{\mathbb{N}}^{+}=\{0,1,2,3,\ldots \}$
Square root	b>0 where b>0
n-th root	for b≠0 and an odd n: $\sqrt[{n}]{a\overset{―}{0}\dot{+}b}=\;\left(+\frac{a}{n\cdot b^{\left(\frac{n-1}{n}\right)}}\;\overset{―}{0}\right)\dot{+}\left(+\sqrt[{n}]{b}\right)$ For b>0 and an even n: $\left(+\frac{a}{n\cdot b^{\left(\frac{n-1}{n}\right)}}\overset{―}{0}\right)\dot{+}\left(+\sqrt[{n}]{b}\right)$, $\left(-\frac{a}{n{\cdot b}^{\left(\frac{n-1}{n}\right)}}\overset{―}{0}\right)\dot{+}\left(-\sqrt[{n}]{b}\right)$

Note: under the inverse operator definition, (SN,+,×) is almost an algebraic field, and if this definition is applied then b=0 as well, and it would be a complete field.

In many cases, the principals of linear algebra, specifically regarding the matrix, are required. Therefore, the soft number field was extended to Soft Logic linear algebra, and as detailed in the next section, this part is required for the current research. Given that a and b are simple scalars (as defined in classic math, a basic component that constructs a vector with dimensions), the soft number $a\overset{―}{1\;}\dot{+\;}b\overset{―}{0}$ is a scalar soft number.

First a soft vector, which is a collection of scalar soft numbers is defined:


$$V=\left(v_{1},v_{2},\ldots ,v_{n}\right)|v_{i}\in SN$$

and based on the soft vector definition, a soft matrix is also defined:

$C=\left\{c_{i,j}|c_{i,j}\in SN\right\}$ when $1\leq i\leq n\;\;,\;1\leq j\leq m\;,\;c_{i,j}=a_{i,j}\overset{―}{0}\dot{+}b_{i,j}$

Like the soft scalar number which has two components, a soft matrix C will also have two components, each constituting a matrix:


$$C=A\overset{―}{1}\dot{+}B\overset{―}{0}=\{\begin{array}{ccc} a_{1,1} & \cdots & a_{1,m_{a}}\\ \vdots & \ddots & \vdots \\ a_{n_{a},1} & \cdots & a_{n_{a},m_{a}} \end{array}\}\overset{―}{1}\;\dot{+}\;\{\begin{array}{ccc} b_{1,1} & \cdots & b_{1,m_{b}}\\ \vdots & \ddots & \vdots \\ a_{n_{b},1} & \cdots & a_{n_{b},m_{b}} \end{array}\}\overset{―}{0}$$

Matrix A, which is a classic math matrix, represents the real part of the soft matrix with the dimensions ($n_{a}\times m_{a}$), while Matrix B, which also is a classic math matrix, represents the soft part of the soft matrix with the dimensions ($n_{b}\times m_{b}$). In other words, the extension of Soft Logic to linear algebra is achieved by simply transforming the coefficients of the soft number components from scalar to matrices.

Let $C_{1}=A_{1}\overset{―}{0}\dot{+}B_{1}$ and $C_{2}=A_{2}\overset{―}{0}\dot{+}B_{2}$. Now, given the above definition, the linear algebra operators can also be applied to soft matrices as described in Table 3:

**Table 3:** Definition of mathematical operators of Soft Logic matrices.

Operator	Definition
Addition	$C_{1}+C_{2}=\left(A_{1}+A_{2}\right)\overset{―}{0}\dot{+}\left(B_{1}+B_{2}\right)$
Multiplication	$C_{1}\cdot C_{2}=(A_{1}\cdot B_{1}+A_{2}\cdot B_{1}overline0\dot{+}B_{1}\cdot B_{2}$

## 4. Representation of management styles with Soft Logic

### 4.1. General approach

The implementation of Soft Logic requires that the evaluation of the final indexes is quantitative, and that the scale is well defined. However, there is no limitation regarding the dimensions and layout of the model. Given the fulfillment of the above requirement, all of the evaluations may be presented using Soft Logic without any data loss (usually due to averaging), including outlier responses as described above, and demonstrated below. According to the Adizes systems, there are four ingredients that form an Individual management style: Producer, Administrator, Entrepreneur, and Integrator, denoted by their first letters: P, A, E, and I, respectively. For generality, let S be the set of styles, and in the Adizes test case, S={P,A,E,I}. Each of these types can be ranked by a four-level discrete scale: Least Preferred, Secondary, Dominant, and Very Dominant, which are denoted $\left[{LP}^{r},\;S^{r}\;,\;D^{r}\;,\;{VD}^{r}\right]$ respectively.

Since the balance between the styles is important in achieving the organization’s goals, both the absolute values of each style, and the tensions between them should be considered. The use of Soft Logic in this regard, enables a representation, and therefore mathematical manipulations of this estimator. For the conveniency of the reader, a list of variables is provided, and explained in the rest of this section:

**Table:** 

S	The set of styles. In the Adizes test case (S={P,A,E,I})
${RgRg}_{s,r}^{\min}$	The lower values of the intervals for each style and rank, where s is the style, and r is the discrete rank (r$=\left\{{LP}^{r},S^{r},D^{r},{VD}^{r}\right\}=\left\{Least\;Preferred,\;\;Secondary\;,\;\;Dominant\;,\;\;Very\;Dominant\right\}$)
${Rg}_{s,r}^{\max}$	Same as ${RgRg}_{s,r}^{\min}$, but indicating the higher value
${CR}_{s}$	The continuous ranks matrix for style s
${{cr}^{\overset{―}{1}}}_{si,sj}$	The type rank component of types si and sj (which forms the matrix ${CR}^{\overset{―}{1}}$)
${{cr}^{\overset{―}{0}}}_{si,sj}$	The tension component between types si and sj (which forms the matrix ${CR}^{\overset{―}{0}}$)
TRy	The threshold for the tension
${CRtr}^{\overset{―}{0}}$	The tension matrix ${CR}^{\overset{―}{0}}$ after applying the threshold TR
${mw}^{typ}$	A major weight for the type ranks (with respect to the tension rank)
${mw}^{ten}$	A major weight for the tension ranks (with respect to the tension type)
$w_{s}$	The weight of type s
or	The overall rank or the rank of an individual

### 4.2. Scaling and indexes

The Adizes questionnaire includes 20 questions, each referring to one subject, e.g., I try to avoid... and four propositions, e.g., long debates, not finishing a job, using numbers and formulas, conflicts with others. The participant is asked to classify the propositions of each subject from the very dominant to the least dominant. Then a score is calculated according to the following scale: Very Dominant – 8 points, Dominant – 4 points, Secondary – 2 points, and Least Preferred – 1 point. This scale follows the original structure of the Adizes methodology and was not modified in this study. The current research applies this scale as part of the standard evaluation procedure defined and validated by Adizes [39]. Since the questionnaire has already undergone reliability and validity testing as part of its original development and has been widely applied in organizational and managerial contexts, no further psychometric validation was required for this study. Therefore, this tool is valid for the current research. Alternatively, instead of ranking the propositions, the participant can mark the scores which leads to the same result. At the end of the questionnaire, $R_{s}$, which is the sum of each of the scores of the first, second, third and fourth propositions, is calculated, yielding the style rank of P, A, E and I, respectively. The rank is set according to a table of ranges, e.g., if the total score for A (Administrator) is between 97 and 104, the style is ranked as Dominant.

The scale that was described above yields four discrete values. However, when evaluating the tension between the styles, this scale may introduce a paradoxical situation. Assume for example that for style A the score was $R_{A}=83$, which falls in the range of 83–96, indicating the rank of Secondary. And also assume that for style E the score was $R_{E}=94$, which falls in the range of 87–94, indicating Dominant. Thus, the tension between Secondary and Dominant, which are consecutive ranks, is one. But actually, the score of A is on the borderline between Least Preferred and Secondary, and the score of E is on the borderline between Dominant and Very Dominant. Thus, it is more accurate to set the tension to a value of two. To overcome this paradoxical situation, the rank was transformed to a continuous one in the following manner. We define the ranking ranges ${RgRG}_{s,r}^{\min}$, ${Rg}_{s,r}^{\max}$, where s is the style (s={Producer, Administrator, Entrepreneur, Integrator}={P,A,E,I}), and r is the discrete rank (r$=\left\{Least\;Preferred,\;\;Secondary,\;Dominant,\;Very\;Dominant\right\}=\{{LP}^{r},S^{r},D^{r},{VD}^{r}\}$). ${RG}_{s,r}^{\min}$ indicates the lower values of the intervals for each style and rank, while ${RG}_{s,r}^{\max}$ indicates the higher values of the intervals for each style and rank. The intervals are defined in the following matrices:


RGs,rmin=  LPr     Sr     Dr     VDr    PAEI(rgP,LPrminrgP,SrminrgP, DrminrgP,VDrminrgA,LPrminrgA,SrminrgA, DrminrgA,VDrminrgE,LPrminrgE,SrminrgE, DrminrgE,VDrminrgI,LPrminrgI,SrminRgrgI,VVDrmin)

and


RGs,rmax= LPr     Sr     Dr     VDr    PAEI(rgP,LPrmaxrgP,SrmaxrgP,DrmaxrgP,VDrmaxrgA,LPrmaxrgA,SrmaxrgA,DrmaxrgA,VDrmaxrgE,LPrmaxrgE,SrmaxrgE,DrmaxrgE,VDrmaxrgI,LPrmaxrgI,SrmaxrgI,DrmaxrgI,VDrmax)

Now, given $R_{s}$, the continuous rank ${CR}_{s}$, which is the rank for style s is given by:


$$\begin{array}{c} {CR}_{s}=\left\{ \begin{array}{ccc} \frac{R_{s}-{rg}_{s,{LP}^{r}}^{\min}}{{rg}_{s,Least\;Preferred}^{\max}-{rg}_{s,Least\;Preferred}^{\min}}+1 & if & \left({rg}_{ss,{LP}^{r}}^{\min}\geq R_{s}\right)\;⋀\;\left(R_{s}\leq {rg}_{s,{LP}^{r}}^{\max}\right)\\ \frac{R_{s}-{rg}_{s,S^{r}}^{\min}}{{rg}_{s,SeconS^{r}}^{\max}-{rg}_{s,S^{r}}^{\min}}+2 & if & \left({rg}_{s,S^{r}}^{\min}\geq R_{s}\right)\;⋀\;\left(R_{s}\leq {rg}_{s,S^{r}}^{\max}\right)\\ \frac{R_{s}-{rg}_{s,\;D^{r}}^{\min}}{{rg}_{s,\;D^{r}}^{\max}-{rg}_{s,\;D^{r}}^{\min}}+3 & if & \left({rg}_{s,\;D^{r}}^{\min}\geq R_{s}\right)\;⋀\;\left(R_{s}\leq {rg}_{s,\;D^{r}}^{\max}\right)\\ \frac{R_{s}-{rg}_{s,\;{VD}^{r}}^{\min}}{{rg}_{s,\;{VD}^{r}}^{\max}-{rg}_{s,\;{VD}^{r}}^{\min}}+4 & if & \left({rg}_{s,\;{VD}^{r}}^{\min}\geq R_{s}\right)\;⋀\;\left(R_{s}\leq {rg}_{s,\;{VD}^{r}}^{\max}\right) \end{array}\right.\; \end{array}$$

where $0.0{\leq CR}_{s}\leq 4.0$

### 4.3. Soft Logic representation

As noted, we would like to represent both the ranks of the styles and the tensions between them. Therefore, we use the component $\overset{―}{1}$ to represent the style rank, and the component $\overset{―}{0}$ to represent the tensions.

For each of these types, the center value is defined as the average of their rank, and is represented by ${CR}^{\overset{―}{1}}$. The rows and the columns of matrix ${CR}^{\overset{―}{1}}$ are formed by the value range of r, and are indexed by si and sj ( si,sj∈{1..4}). Consequently, si and sj are the index value of r. Naturally, the diagonal represents the average of each rank value itself, namely the rank of this value. The values above the diagonal are the combination s of the rank of both types, and as defined is calculated by the average $\left(\;\frac{cr+{cr}_{sj}}{2}\;\right)$. Since $\forall si,sj\;:\;\left(\;\frac{{cr}_{si}+{cr}_{sj}}{2}\;\right)=\left(\;\frac{{cr}_{sj}+{cr}_{si}}{2}\;\right)$, the matrix is triangular, and the values below the diagonal are discarded. Thus, ${CR}^{\overset{―}{1}}$ forms the component $\overset{―}{1}$.


$${CR}^{\overset{―}{1}}=\left(\;\frac{{cr}_{si}+{cr}_{sj}}{2}\;\right)=\begin{array}{cc} & \begin{array}{cccc} P & A & E & I \end{array}\\ \begin{array}{c} P\\ A\\ E\\ I \end{array} & \left(\begin{array}{cccc} {cr}_{P} & \;\cdots & \cdots & \frac{{cr}_{I}+{cr}_{P}}{2}\\ & \ddots & \vdots & \vdots \\ & & \ddots & \ddots \\ & & & {cr}_{I} \end{array}\right) \end{array}$$

The tensions between the styles are defined as the absolute difference between them, and are represented by ${CR}^{\overset{―}{0}}$. The indexes of ${CR}^{\overset{―}{0}}$ are the same as ${CR}^{\overset{―}{1}}$. The diagonal is the difference between each type rank itself, and therefore is zero. The values above the diagonal are the absolute differences between the ranks of both types, and are calculated by the difference $\left|{cr}_{si}-{cr}_{sj}\right|$. Again, since $\forall si,sj\;:\;\left|{cr}_{si}-{cr}_{sj}\right|=\left|{cr}_{sj}-{cr}_{si}\right|$, the matrix is triangular, and the values below the diagonal are discarded. Thus, ${CR}^{\overset{―}{0}}$ forms the component $\overset{―}{0}$.


$${CR}^{\overset{―}{0}}=\left(\;\left|{cr}_{si}-{cr}_{sj}\right|\;\right)=\begin{array}{cc} & \begin{array}{cccc} P & A & E & I \end{array}\\ \begin{array}{c} P\\ A\\ E\\ I \end{array} & \left(\begin{array}{cccc} 0 & \;\cdots & \cdots & \left|{cr}_{I}-{cr}_{P}\right|\\ & \ddots & \vdots & \vdots \\ & & \ddots & \vdots \\ & & & 0 \end{array}\right) \end{array}$$

The above definitions represent the pure mathematical model. Nonetheless, in practical terms some differences may be insignificant. Therefore, a threshold TR is added, so that when ${CR}^{\overset{―}{0}}\leq TR$, ${CR}^{\overset{―}{0}}$ will be zeroed. The new definition of ${CR}^{\overset{―}{0}}$ with the threshold applied, which is denoted ${CRtr}^{\overset{―}{0}}$, will be:


$${CRtr}^{\overset{―}{0}}=\left(\;\left\{ \begin{array}{cc} \left|{cr}_{si}-{cr}_{sj}\right| & \left|{cr}_{si}-{cr}_{sj}\right|>T\\ 0 & otherwise \end{array}\right.\;\;\right)=$$

$$\begin{array}{cc} & \begin{array}{cccc} P & A & E & I \end{array}\\ \begin{array}{c} P\\ A\\ E\\ I \end{array} & \left(\begin{array}{cccc} 0 & \;\cdots & \cdots & \left\{ \begin{array}{cc} \left|{cr}_{I}-{cr}_{P}\right| & \left|{cr}_{I}-{cr}_{P}\right|>T\\ 0 & otherwise \end{array}\right.\;\\ & \ddots & \vdots & \vdots \\ & & \ddots & \vdots \\ & & & 0 \end{array}\right) \end{array}$$

Note that when TR=0, i.e., there is no threshold, ${CR}^{\overset{―}{0}}={CRtr}^{\overset{―}{0}}$, and therefore, ${CR}^{\overset{―}{0}}$ is an isolated case of ${CRtr}^{\overset{―}{0}}$.

The overall comprehensive management type of a person is given by the soft number:


$$Type={CR}^{\overset{―}{1}}\overset{―}{1}\;\dot{+}\;\;{CRtr}^{\overset{―}{0}}\overset{―}{0}$$

### 4.4. Weighting and order

In classical mathematics, the order between numbers is clear, and in Soft Logic the order between soft zeros is also clear. However, the order between two soft numbers is unclear, which is the outcome and the representation of the dilemma described above.

To address this issue in reference to the current research, we define two major indexes:

a) Let TY be the vector of the ranks of each type. This vector is actually the diagonal of the matrices ${CR}^{\overset{―}{1}}$, therefore, $TY={{cr}^{\overset{―}{1}}}_{i,i}\;\;\;(\forall i\in \left\{1.\mathrm{.4}\right\})$.
b) Let tn be the overall tension between the types. Unlike the type rank, in the tension case we are interested in small values, therefore, to reverse the order, the calculated tensions will be reduced from the maximal tension value $t^{\max}$. The definition of tn is:

$$tn=t^{\max}-\sum_{\forall i\neq j}\left|{cr}_{i}-{cr}_{j}\right|=\frac{\left|{cr}_{P}-{cr}_{A}\right|+\left|{cr}_{P}-{cr}_{E}\right|+\left|{cr}_{P}-{cr}_{I}\right|+\left|{cr}_{A}-{cr}_{E}\right|+\left|{cr}_{A}-{cr}_{I}\right|+\left|{cr}_{E}-{cr}_{I}\right|}{6}$$

note: if a threshold is introduced, CR should be replaced with cRtr.

Now, two sets of weights are defined:

a) A major weighting between the type rank and the tension rank, which has two components, ${mw}^{typ}$ and ${mw}^{ten}$, respectively (${mw}^{typ}\leq 1,\;\;{mw}^{ten}\leq 1,\;\;{mw}^{typ}+{mw}^{ten}=1$)
b) An internal set of weights for each type $w_{s}\;(\sum_{\forall s}\;w_{s}=1)$.

The overall rank or of an individual will be:


$$or=\left({mw}^{typ}\cdot \sum_{i\in \left\{1.\mathrm{.4}\right\}}w_{i}\cdot {ty}_{i}\right)+\left({\;\;mw}^{ten}\cdot tn\right)$$

## 5. The empirical study

### 5.1. Framework

The major aim of the empirical study was to demonstrate how the methodology of representing management skills with Soft Logic can be carried out. To this end, we created a questionnaire that included three parts: a) general information and consent; b) demographic information; c) the Adizes questionnaire.

The questionnaire was conducted on the Qualtrics online survey platform. The questionnaire was distributed among executives and project managers at various levels within both public and private organizations, and across different sectors. The distribution was carried out digitally through the local branch of the PMI (Project Management Institute) organization [48], a professional organization specializing in cutting-edge project management practices. The branch members include managers from diverse ranks in leading high-tech and traditional industry organizations. Because the answers may contain sensitive personal data, the questionnaire was completely anonymous (only quasi-information such as demographic data was collected), and participation was on a voluntary basis. The questionnaire was reviewed and approved by the ethics committee of the Faculty of Engineering of Ariel University (No. of Approval: AU-ENG-SK-20240417). The information provided by the participants during the distribution of the questionnaire is stored in a secured, encrypted manner, with access restriction services provided by the researcher’s institution.

### 5.2. Participants

The recruitment period started on 21/04/2024 and ended on 20/05/2024. Overall, 59 people participated in the empirical study. However, after disqualifications only n=40 participants with valid answers remained. Disqualification was carried out mainly due to incompletion of the task. 25% of the participants were 31–40 years old, 37% 41–50 years old, 20% 51–60 years old, and 18% were over the age of 60. About 70% of the participants were males, and 30% were females. 35% of the participants had an undergraduate degree, 58% a graduate degree, and 7% did not have an academic degree. 73% were employees, 25% self-employed, and 2% were unemployed. In reference to the role of the participants who were employees, 69% were managers, 8% management consultants, 5% engineers, and 18% served different roles such as system analysis, and inspection. The sample comprised individuals holding practical managerial responsibilities across various organizational tiers. These included project managers, department heads, and lower-level managers who were actively involved in planning, coordination, and decision-making processes within their respective organizations. 87% of the participants had seniority of over 10 years, 5% of 6–10 years, and 8% of 1–5 years. This population was intentionally selected to reflect the practical and diverse applications of management styles in everyday organizational contexts, particularly at the operational and mid-management levels.

### 5.3. Representation of the management types with Soft Logic

**Fig 1:**
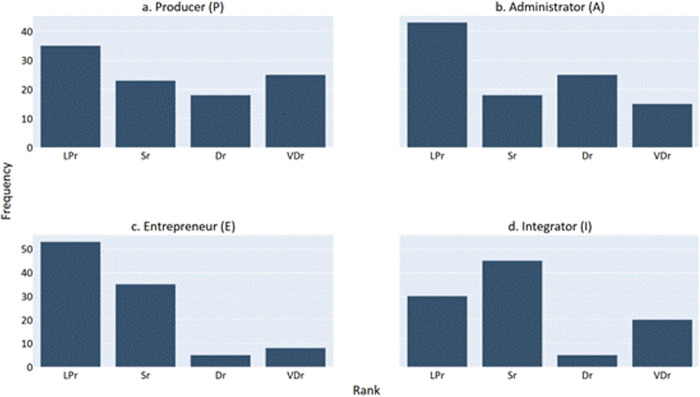
The distribution of the ranks for each management type. The values ${LP}^{r},S^{r},D^{r},{VD}^{r}$ represent  Least Preferred,  Secondary, Dominant, and Very Dominant, respectively.

The score for each type S={P,A,E,I} was calculated according to the Adizes evaluation systems, and then each type was discreetly ranked according to the values $r=\{{LP}^{r},S^{r},D^{r},{VD}^{r}\}$ by the provided intervals. The distributions of the ranks for each type across the entire empirical study population is depicted in Fig 1.

**Fig 2:**
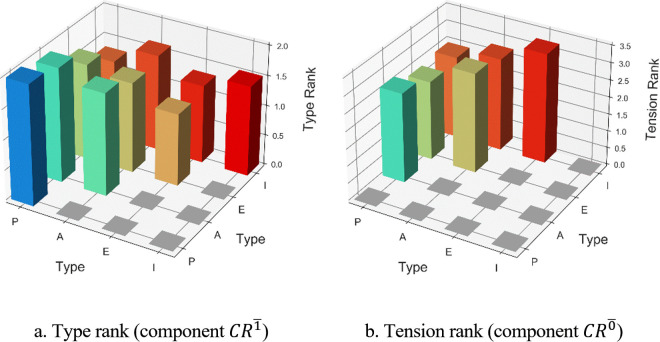
The distributions of the components of the Soft Logic number that represent the management rank. **(a)** Type rank (component ${CR}^{\overset{―}{1}}$) **(b)** Tension rank (component ${CR}^{\overset{―}{0}}$).

Then, the ranks were transformed to a continuous sequence of values as described in the methodology section, and the components of the Soft Logic number, ${CR}^{\overset{―}{1}}$ and ${CR}^{\overset{―}{0}}$, which represent the types’ ranks and the tensions could be calculated straightforward. The maximal tension value was $t^{\max}=4$. In this case we applied a threshold of TR=0, i.e., no threshold. The distributions of the components of ${CR}^{\overset{―}{1}}$ and ${CR}^{\overset{―}{0}}$ are illustrated in Fig 2. In the type rank graph (Fig 2a), both the X-axis and the Y-axis represent the types, while the Z-axis represents the type rank, and identically to the matrix ${CR}^{\overset{―}{1}}$ it is triangular. Values on the diagonal of the X-Y plane represent the rank of a specific type, values beyond this diagonal (in the far end of the figure) represent the combined average ranks of two types, and the values below this diagonal (in the close end of the figure) are presented in gray because they are mirrored. In the tension rank graph (Fig 2b), both the X-axis and the Y-axis represent the types, the Z-axis represents the tension rank, and identically to the matrix ${CR}^{\overset{―}{0}}$ it is triangular. Values on the diagonal of the X-Y plane represents the tension between a type and itself, therefore must be zero and appears in gray. Values beyond this diagonal (in the far end of the figure) represent the tension between the two types, and the values below this diagonal (in the close end of the figure) also appear in gray because they are mirrored.

### 5.4. Results

**Fig 3:**
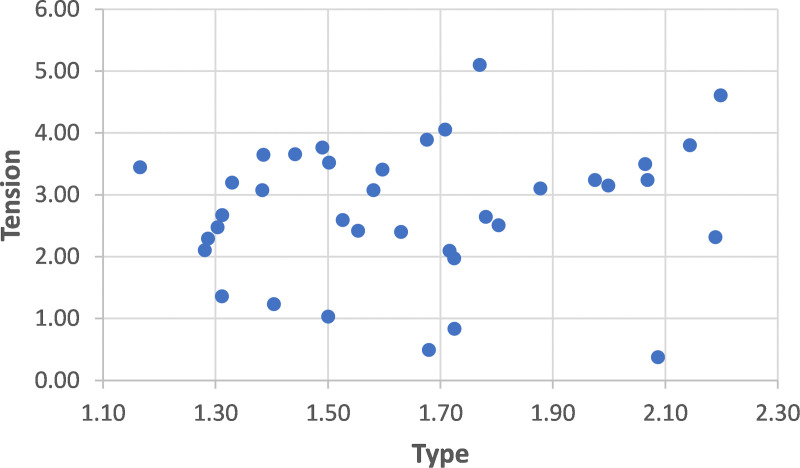
The full picture of the participants’ ranks (after anonymization with random noise).

For each participant we received a specific Type rank component (${CR}^{\overset{―}{1}}$) and Tension rank component (${CR}^{\overset{―}{0}}$). For demonstration purposes, we defined the internal set of weights ($w_{s}$) to be equal for each type, i.e., $w_{P}=w_{A}=w_{E}=w_{I}=0.25$. The full picture of the participants regarding their overall type rank and overall tension rank can now be drawn by means of an XY plot. However, part of the empirical study ethics conditions were that the data be published based solely on a statistical basis and the data of a specific individual will not be disclosed. Therefore, we used one of the common techniques to preserve the privacy of the dataset by applying random perturbation [49]. We added random noise to the results of the XY plot, with a NORMAL distribution function of μ=0. Since the distribution is symmetric around the zero value, the overall picture remains, but the specific data is hidden (to preserve the privacy of the participants). The XY plot after randomization is depicted in Fig 3. Each participant is represented by a dot, where the X-axis illustrates the type rank and the Y-axis portrays the tension rank. While this map shows the detailed ranks, it also provides a visualization of the dilemmas, for example, a candidate with a higher type rank and a lower tension rank vs. a candidate with a lower type rank and a higher tension rank. This map, may support the decision-making process as explained below.

**Fig 4:**
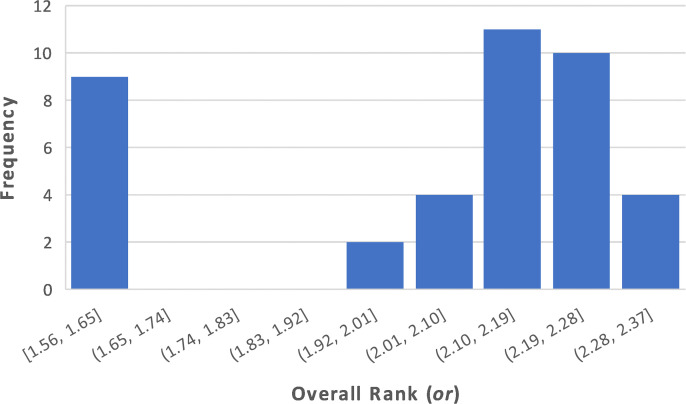
The distribution of the overall score (or).

Now, the overall rank or can be calculated. We used major weighting between the type rank and the tensions of ${mw}^{typ}=0.6$, and ${mw}^{ten}=0.4$. In reality, these weights should be set by the stakeholder, e.g., a manager in the organization according to the relevant preferences. The distribution of the overall score (with the abovementioned major and internal weights, but without anonymization with random noise) is depicted in Fig 4.

These results, which demonstrates an empirical study on a specific population, may serve as an initial ‘map’ that can be provided to a decision-maker regarding the promotion/ recruitment for a managerial role. First, Fig 4 illustrates how this population varies according to the managerial type, and may support initial decisions. For example, a case in which all, or almost all values are concentrated on the lower side, may suggest that there are no candidates who satisfy the requirements. Or for instance, when the values are dense, the decision might be difficult, and more supporting parameters might be required. Second, screening the map in Fig 3 may indicate the specific individuals who stand out, and can be transferred to the final stage of the decision.

It is important to note that the weighting parameters used in this section were selected solely for demonstration purposes. In practical applications, the weights—both the internal ones assigned to each managerial type (e.g., Producer, Administrator, Entrepreneur, Integrator) and the major weights balancing type rank and tension—should be determined based on the specific organizational needs and role requirements. For example, in a high-tech company that emphasizes innovation, agility, and risk-taking, greater weight might be assigned to the Entrepreneur (E) style. In contrast, in more structured or compliance-driven environments such as government agencies or manufacturing sectors, the Administrator (A) style may be weighted more heavily. This flexibility allows decision-makers to adapt the tool to various managerial contexts, making it not only diagnostic but also strategically actionable.

## 6. Discussion

Over the past few decades, leadership and management styles have emerged as a central theme in management research, drawing significant attention among scholars and practitioners alike. For example, Anderson and Sun [50] focus on addressing the ongoing issues in leadership style research, and identified four major problems: (1) the lack of consensus on the number of distinct leadership styles; (2) the significant overlap between styles, both conceptually and empirically; (3) the contested dimensionality of these styles; and (4) the need for a comprehensive hierarchical model to classify leadership behaviors. An additional example is the study conducted by Zhang et al. [51] who explored the concept of paradoxical leadership and its impact on employee creativity through the lens of subjective ambivalence. Generally, it has shown that managers have some uniqueness compared to non-managers, e.g., in the decision-making process under uncertainty or when negotiating [52]. The researchers placed particular emphasis on how employees’ holistic thinking serves as a moderating factor in these relationships. Like these studies, which emphasize the complexity of leadership behaviors and their effects on organizational outcomes through advanced assessment methods, the current study seeks to represent the multifaceted nature of management behaviors, enabling a better exploration of this skill. It focuses on the Adizes Management Style Questionnaire [21], which is similar to the paradoxical leadership presented by Zhang et al. [51], or the hierarchical structure to organize leadership styles used by Anderson and Sun [50], that evaluate multiple management styles which may present tensions between tyles, resulting in conflicting or overlapping characteristics.

By applying Soft Logic, the current study offers a framework to represent these tensions and thus provides a comprehensive approach to the evaluation task of managerial styles, adding depth to the traditional models of management assessment. While existing assessment tools often evaluate managerial styles in isolation, this approach enables a more in-depth representation of the tensions and interplay between the different styles within a specific individual. By doing so, it does not simply categorizing management styles but rather captures the dynamic relationships and conflicts between them, offering a richer representation framework. Specifically, in the existing literature, the PAEI framework introduced by Adizes [21] has been widely used to classify management styles into four categories: Producer, Administrator, Entrepreneur, and Integrator. However, this framework tends to treat each style as distinct and separate, which may oversimplify the complexities of leadership in real-world contexts. The current study builds on this framework by using Soft Logic to represent not only the individual styles but also the inherent tensions between them.

Previous research, such as that of Hirschprung, Klein, and Maimon [42] demonstrated the utility of Soft Logic in addressing paradoxical situations, specifically in the context of the privacy paradox. Their study used Soft Logic to model the gap between users’ privacy concerns and their actual behavior in the digital environments, highlighting the contradiction between what users claim regarding privacy and how they act [53]. Their study serves as a foundation for extending the application of Soft Logic beyond digital privacy to complex human behaviors in leadership and management, as demonstrated in the current research. The Soft Logic framework applied here allows us to assess managerial styles relating to both dominant traits and the tensions between different management styles which can be paralleled to a paradoxical situation. In the managerial case, the tension is not really a paradox, however, its existence introduces a dilemma identical to the privacy paradox case.

A key contribution of this study is the ability to use Soft Logic to assess both the strengths and tensions between different managerial styles. This comprehensive representation provides organizations with a deeper understanding of their leaders’ behaviors, enabling more informed decision-making in areas such as recruitment, leadership development, and team composition.

However, it is important also to acknowledge the subjective elements inherent in this methodology. The weightings, both the major one between the type rank and the tensions, and the internal one for each type of managerial style (Producer, Administrator, Entrepreneur, and Integrator), introduce a degree of subjectivity. These weightings can be adjusted to meet specific organizational requirements, which adds flexibility but may also introduce bias if not carefully considered. Nonetheless, this adaptability allows organizations to tailor the evaluation process to align with their unique leadership needs and strategic objectives. Further research may offer a more methodological approach to set these weights.

Despite its contributions, the study is subject to several limitations. First, the sample size was relatively small which may limit the generalizability of the findings. The sample primarily consisted of managers with a seniority of above 10 years and management consultants. However, the main purpose of the empirical study was to demonstrate how the methodology can be applied, and not to review managerial skills. Future studies can aim to include larger and more diverse samples, encompassing managers from various organizational levels and sectors (e.g., public, private, non-profit). A broader sample would provide a more comprehensive understanding of how the Soft Logic approach can be applied across different organizational settings. Another limitation is the explainability of this model, whereby a decision-maker who does not possess the necessary mathematical skills may find difficult to comprehend. To overcome this obstacle perhaps a transformer can be developed in another study, to enable access to the mathematical results in a more user-friendly way.

While this study focuses on the Adizes management styles framework as a demonstrative case, the proposed methodology is not inherently limited to this model. The Soft Logic–based approach is designed to be adaptable to a variety of multi-dimensional evaluation tools, including contemporary leadership frameworks such as transformational–transactional leadership, servant leadership, or strategic agility models. Moreover, the method may be extended beyond managerial or leadership assessment, and potentially can be applied in other domains that involve decision-making with complex trade-offs between competing characteristics, such as, project management, and talent development frameworks. Adopting the current proposed methodology to research in these areas could demonstrate how to capture the complexities of other multi-dimensional processes, e.g., in Quality Function Deployment (QFD), which translates customer needs into prioritized improvement actions. Dror and Sukenik’s QFD-based framework emphasizes the systematic addressing of diverse dimensions, a principle that may be mathematically expressed by the Soft Logic model [54]. Extending this methodology to other domains may offer structured solutions to complex organizational challenges, such as integrating customer priorities with management styles and leadership strategies. Another aspect that may be investigated in future research could be how organizations and individuals navigate the inherent tension between effectiveness – defined as achieving necessary results; and efficiency – defined as performing activities with minimal waste [39]. As mentioned above, such research could also focus on developing user-friendly software tools that will enable efficient and accurate application without requiring advanced mathematical expertise. The interface should be tailored to the user’s level of literacy, ensuring it is adapted to the specific requirements of each domain. Moreover, considering a timeline, future research could apply the proposed methodology to follow managers over time, to examine how their leadership styles evolve given the changing organizational needs and challenges.

While the Adizes Questionnaire assesses managerial styles, subsequent studies could investigate a more specific aim, for example, employing balance resource mobilization for immediate outcomes with energy conservation for long-term sustainability. By examining this trade-off across various organizational contexts, researchers could determine whether particular management styles and the level of tensions are more suited to certain types of decision-making. Another direction for future studies is to assess how different managerial styles influence the balance between short-term expediency and long-term planning. Decisions that might seem effective and efficient in the short term could lead to long-term vulnerabilities. A longitudinal study could examine how different leaders (e.g., Entrepreneur or Integrator vs. Administrator and Producer) approach crises and change management, particularly focusing on the trade-offs between immediate resolutions and the sustainability of their decisions. The mathematical representation offered by the current research, provide the model’s foundations for such researches. An additional direction for future research involves examining the predictive validity of the proposed tool. Specifically, it would be valuable to assess whether the representation of tensions between managerial styles, as captured by the Soft Logic model, can serve as a predictor of actual managerial performance. For example, the tool could be applied during the screening process for managerial roles, and the candidates’ subsequent job performance within the organization could be monitored and evaluated. Such an empirical validation would support the tool’s practical implementation in organizational decision-making contexts and strengthen its relevance beyond diagnostic applications.

Another promising direction for future research involves integrating the organizational life cycle stage into the assessment process. According to the Adizes methodology, organizations progress through distinct stages such as infancy, childhood, and maturity, each characterized by different managerial challenges and needs [21]. Evaluating managerial styles in light of an organization’s current life cycle stage may reveal patterns of alignment or misalignment between managerial tendencies and organizational demands. Such an approach could enhance the contextual accuracy of the tool and provide deeper insights into the dynamic interplay between leadership profiles and organizational development.

It is important to note that the aim of this study was not to characterize the empirical study’s managerial population per se, but to develop and demonstrate a comprehensive evaluation methodology for managerial styles. The proposed tool is intended for broad applications, including recruitment and promotion. Consequently, it may be relevant not only for currently acting managers, but also for those being considered for managerial roles.

In conclusion, this study offers a comprehensive approach to the representation and evaluation of managerial styles, highlighting the dynamic tensions between different leadership behaviors, and considers the crucial balance between them. By integrating Soft Logic with the Adizes Management Style Questionnaire, the study provides a flexible and multi-dimensional tool for representing management styles in complex organizational environments. The findings contribute to both the academic literature and practical applications, offering organizations a deeper understanding of their leaders and the interactions between their managerial styles.

Beyond its theoretical contribution, the proposed methodology can be applied as a practical decision-support tool in organizational contexts, which can be implemented through a structured multi-stage process. Namely, first the organization defines the managerial role based on its current characteristics, strategic goals, and cultural environment (e.g., a startup emphasizing innovation may seek a different leadership profile than a regulatory agency prioritizing process stability). Second, the relevant weights for each managerial style and for the type–tension balance are determined accordingly. Third, candidates are evaluated using the Soft Logic–based framework, allowing a nuanced representation of their leadership profiles. Finally, decision-makers can make use of the output in order to provide a foundation for their decisions concerning promotions or recruitment, based on the degree of alignment between the candidate’s profile and the role requirements.
